# “It’s where learning and teaching begins ‒ is this relationship” — insights on the teacher-student relationship at university from the teachers’ perspective

**DOI:** 10.1007/s10734-022-00867-z

**Published:** 2022-05-20

**Authors:** Gerda Hagenauer, Franziska Muehlbacher, Mishela Ivanova

**Affiliations:** School of Education, Department of Educational Science, School Research and School Practice, Paris Lodron University of Salzburg, Erzabt-Klotz-Straße 1, 5020 Salzburg, Austria

**Keywords:** Teacher-student relationship, Higher education, Professional role, Closeness, First-year students

## Abstract

Positive teacher-student relationships have been identified as important for teacher and student well-being and for high-quality teaching and learning processes and outcomes. However, research on the perceptions of teachers in higher education on a high-quality relationship with students and the perceived antecedents is still scarce. This study aimed to address this research gap by interviewing 15 Australian higher education teachers about their perception of forming relationships with first-year students. The results suggest that the quality of the teacher-student relationship comprises both a professional and an interpersonal dimension, reflecting the different roles teachers and students assume within it. These two dimensions can be further differentiated into various relational quality indicators, such as approachability, care, support, trust, and others. Furthermore, the results indicate that several contextual and personal attributes contribute to the development of this relationship. Implications about how to shape positive relationships between teachers and students in higher education are discussed.

## Introduction

In 1995, Baumeister and Leary proposed the “belongingness hypothesis”, which states that “human beings are fundamentally and pervasively motivated by a need to belong, that is, by a strong desire to form and maintain enduring interpersonal attachments” (p. 522). A large body of empirical findings, resulting mainly from Self-Determination Theory (Deci & Ryan, 2002) and Attachment Theory (Cassidy & Shaver, 2008), support this statement within educational contexts. In this study, we focus on the higher education (HE) context and on one particular significant relationship within that setting: the teacher-student relationship (TSR). Research has repeatedly revealed that meaningful relationships between students and teachers in HE provide several benefits. These benefits have been mainly explored from the students’ perspective, while they are less frequently researched from the perspective of HE teachers. For example, studies have shown that a strong TSR is positively related to students’ motivation, engagement, achievement, retention, and well-being (e.g. Eloff et al., 2021; Kim & Lundberg, 2016; Leenknecht et al., 2020; Richardson & Radloff, 2014; Snijders et al., 2020; Xerri et al., 2018). These findings align well with Tinto’s model on student dropout (1975), which proposes that, along with academic integration, social integration also functions as an important antecedent to prevent students from dropping out of university.

Thus, the establishment of positive TSRs must be regarded as a significant educational aim. However, due to the massification of HE (Hornsby & Osman, 2014), accompanied by an increased student-staff ratio and an increased emphasis on research output and performativity (Wilson & Holligan, 2013), the development of meaningful TSRs occurs under complicated and eventually unconducive conditions that may result in disadvantages, such as a decrease in the well-being of HE teachers. These changes are also observable in the Australian HE sector (Richardson & Radloff, 2014), the context in which the present study was conducted.

Nevertheless, given that a positive TSR contributes to student satisfaction with HE (Snijders et al., 2020) and, more basically, functions as a core foundation for the establishment of a stimulating learning climate (pedagogic perspective, e.g. Regan, 2012), researchers continue to examine TSRs in HE, and evidence of their value steadily increases. Still, as Hagenauer and Volet (2014) and Karpouza and Emvalotis (2019) claimed, TSRs must yet be considered a neglected research agenda in HE. This is particularly evident as regards the question of what constitutes and fosters a high-quality TSR. While some research has explored students’ expectations of teaching in HE, including their expectations about TSRs (e.g. Holmes et al., 1999), the perspective of HE teachers has yet to be explored (Asikainen et al., 2018).

Relatedly, many researchers who highlight the importance of TSRs for high-quality teaching and learning call for professional development of HE teachers in terms of enhancing their interpersonal skills (Asikainen et al., 2018; Grantham et al., 2015). However, if we lack knowledge about what contributes to the establishment of positive TSRs and insights about the constitutive elements, the conceptualization of such professional development courses may be difficult, and it may lack the theoretical and empirical grounding that focuses on the specifics of TSRs in the context of HE teaching and learning.

Taking into account the need for more research into TSRs in HE in general and into the perspective of HE teachers in particular, the present study offers insights into the perceptions of HE teachers on the formation and constitutive elements of meaningful TSRs. The focus is placed on relationships with first-year students, as the transition to university is an emotional phase (Hazel et al., 2008) in which positive affective bonds with faculty are of particular relevance in fostering successful adaptation and integration into the new social environment (Lay-Hwa Boden, 2013).

### On the conceptualization of TSRs in HE

A clear and shared definition and conceptualization of TSRs in HE is still missing, which hampers scientific theorising and restricts the comparability of empirical results. Though this limitation regarding a homogenous understanding of TSRs exists, there is some agreement on relevant features of such relationships. More concretely, researchers argue (Hagenauer & Volet, 2014; Snijders et al., 2018, 2020, 2021) that a TSR must be understood as a multidimensional concept that comprises different facets. In this research, we rely on the conceptualization provided by Hagenauer and Volet (2014), who distinguish between two subordinate dimensions, namely the affective and the support dimensions. More recently, Hagenauer et al., (2016) refer to the “interpersonal” and “professional” dimensions when relating to the affective and support dimension of TSRs. This differentiation considers that teachers and students relate to each other on different levels based on the roles they fulfil (Ei & Bowen, 2002), which are necessarily strongly intertwined. Supporting this distinction, Dicker et al., (2019) claimed that “learning and teaching are both deeply personal as well as professional activities, and it is unsurprising that relationships formed in the classroom impact upon learning” (p. 1432).

Furthermore, a TSR must be understood as an adult-adult relationship that is still inherently hierarchical (Karpouza & Emvalotis, 2019), with a power advance in favour of the teacher. However, within this power-relationship, HE teachers and students have a wide scope of how to form the relationship as, generally, each relationship is characterised by mutuality. For instance, HE teachers can decide to flatten the hierarchy, for example by applying a whole-class approach that is more student-centred and considers students’ voices and opinions in terms of relevant course-related issues (Bovill, 2020). Students, in turn, have to take on a higher responsibility for their studies. Thus, although a TSR always has a power component, the amount of power that is enacted within the concrete classroom and within out-of-classroom interactions is partly dependent on the HE teacher and his/her view of the nature of TSRs and learning partnerships and partly on the students’ views or expectations regarding this relationship.

Relatedly, addressing the malleability of TSRs, the dynamic, processual, and reciprocal character of such relationships needs to be outlined (Karpouza & Emvalotis, 2019). Through various formal and informal interactions that can be experienced either positively, neutrally, or negatively, relationships develop over time and are in a constant state of flux.

Finally, if we address TSRs in HE, we need to consider that the understanding of a high-quality TSR is strongly context-bound. In particular, cultural-educational differences need to be taken into account, suggesting that the cultural-educational context with its underlying values and norms significantly influences the perception of what constitutes a high-quality TSR (Volet, 2001). More concretely, differences between cultures (e.g. in terms of the individualistic vs. collectivistic distinction), institutions (e.g. research-focused vs. applied HE institutions), domains (e.g. hard vs. soft sciences), and factors of the individual (e.g. gender, first-generation student, socio-economic background, undergraduate vs. graduate and postgraduate student, international student vs. domestic student, and personality) are likely to influence how relationships are formed between students and teachers and what is perceived as appropriate and a quality indicator within these relationships. With regard to culture, for instance, Hsieh (2012) has shown that teachers from China understand TSRs differently compared to British teachers. Likewise, in a comparative study of Australian and German HE teachers, Hagenauer et al. (2016) found that Australian teachers enacted the interpersonal dimension of a TSR in a more pronounced way compared to their German counterparts.

In conclusion, studies on TSRs have to take into account that relationships always result from an interaction of at least two people, underscoring its reciprocal and dynamic nature that is shaped by the situation and the broader context. Within the HE context, these relationships are hierarchical, but the teachers and students can determine the way the hierarchy is lived out. Finally, a TSR is multidimensional, comprising not only an interpersonal component, but also a professional (pedagogical) one.

### Relational dimensions of TSRs — empirical findings

Many studies have been conducted on the frequency of student–teacher interactions in HE. Some of those studies have demonstrated that students do not experience interactions with university staff very frequently, and if they do, the staff evaluates these interactions more positively than the students (see Richardson & Radloff, 2014 for an AUS sample). Moreover, the findings of Kim and Lundberg (2016) have shown that minority students in the USA reported less frequent interactions with their HE teachers than non-minority students. The same can be said for international students with lower financial backgrounds and lower academic preparedness (Glass et al., 2015 for an US sample). Dobransky and Frymer (2004) point to the importance of out-of-class interactions for TSRs. Although the frequency of interactions proved to be an important influencing factor leading to the positive outcomes of various students, the quality of these interactions proved to be of greater importance.

As relationship theory suggests, interactions must not be equated with relationships; interactions build the basis for the establishment of TSRs (Baumeister & Leary, 1995). The question that follows is how a positive relationship can be described through concrete indicators that go beyond the broad distinction of “high quality vs. low quality”. Previous research has already detected some of these relational indicators, which can be characterised by approachability, availability, enthusiasm, power, trust, respect, openness, fairness, authenticity/transparency, benevolence, rapport, closeness (as a synonym to intimacy or immediacy) and care (e.g. Anderson et al., 2020; Denzine & Pulos, 2000; Dicker et al., 2019; Dobransky & Frymier, 2004; Eloff et al., 2021; Karpouza & Emvalotis, 2019; Strachan, 2020). However, most of the studies were conducted from the students’ perspective and leave the lecturers’ perspective mainly unilluminated.

“Closeness” as an indicator of the interpersonal component of a TSR is discussed critically due to the boundaries that have to be maintained, and thus, deserves special attention. As the study results of Ei and Bowen (2002; USA) reveal, a majority of HE students perceive sexual and romantic relationships, doing favours for the faculty (e.g. giving the instructor a ride home), and doing activities with faculty alone (e.g. having lunch with the professor) as inappropriate interpersonal behaviour (see also Holmes et al., 1999), while having out-of-class communication with lecturers within a group of students and using first names in interactions is perceived as appropriate. Interestingly, ratings of appropriateness of behaviour differed between groups of students, revealing that female students were more critical of inappropriate behaviour than were male students.

Nonetheless, if the interpersonal relationship is kept within the boundaries perceived as appropriate, it seems to serve several advantages for students. In that regard, Snijders et al. (2020) showed that affective commitment (as an indicator of closeness) was the strongest predictor of student engagement and outperformed the more professional-related components of TSRs.

In addition, Lay-Hwa Boden’s (2013) results point out that close and supportive relationships, as reflected for instance in disclosure, not treating students as numbers, listening to students’ concerns, building rapport with them and answering their emails (as part of the lecturers’ approachability and availability), are of utmost importance for first-year students. She further argues that positive TSRs enhance students’ confidence and lessen the transition shock; most importantly, they impede detachment from the university from the very beginning.

### The present study — research questions

The literature review has pointed out that TSRs in HE are extremely important. Thus, it is imperative to foster research attempts to understand what constitutes a high-quality relationship and which factors contribute to such a relationship. In doing so, in this study, we bring the HE teachers’ perspective, which has been neglected so far in favour of the students’ perspective, to the fore. Furthermore, we specifically address TSRs with first-year students, who are particularly vulnerable during the transition phase to university and may benefit particularly strongly from a meaningful TSR. In this regard, the present study pursues the following two main research questions:

- RQ 1: Which are the core relational indicators of a high-quality TSR with first-year students from the HE teachers’ perspective?
- RQ 2: Which personal and contextual factors contribute to the establishment of a TSR from the HE teachers’ perspective?

## Method

### Participants and interviews

The study followed an exploratory qualitative research approach. The participants were fifteen experienced university teachers (six males and nine females) teaching students majoring in teacher education at two public Australian universities. The participating teachers had many years of teaching experience in teacher education and taught a cross-section of subject areas within that discipline (e.g. science education, literacy education, curriculum planning). All of them had experience in teaching first-year classes. Twelve of the HE teachers were lecturers in teacher education (e.g. associate lecturer, senior lecturer; mostly at the postdoctoral level), and three of them were Associate Professors in Education, with broader teaching and research responsibilities. Five of the participants were international faculty, but had already some years of teaching experience in Australia.

Two rounds of in-depth, semi-structured interviews were conducted in the study year 2012. One interview took place prior to the start of the academic year and the second several weeks into the first semester. While the first interview covered the teachers’ reflections on TSRs in general, the second focused on the development of relationships with students based on concrete interactions while teaching one of their current classes. As not all teachers were teaching first-year students that semester, only nine teachers participated in the second interview (four males and five females).

The main interview questions were:

- *Interview 1*: (1) Quality of TSR: Which characteristics or attributes describe a good or “ideal” relationship between first-year preservice teachers and their teacher in a seminar group? (2) Antecedents: Can you think of some factors that you consider as facilitating and others, that you consider as impeding for the shaping of a positive relationship?
- *Interview 2*: What comes to mind when you think about the relationship you’ve built with that group of students and with particular students in this group?

Each main question was followed by several prompts and probing questions to get a rich understanding on the HE teachers’ perspective and experiences.

Each interview lasted between 35 and 75 min, averaging roughly 50 min. Interviews were audiotaped and transcribed verbatim. Before the interview took place, permission from the ethics committee was obtained. In addition, written informed consent was given by the participants.

### Data analysis

All transcripts were analysed using structuring qualitative content analysis (Mayring, 2015). First, the interviews were read through to obtain a broad idea of which codes were inherent in the data material. Next, a deductive-inductive category system was created, which included two broad categories: *Indicators of TSRs* and *Antecedents of TSRs*. Furthermore, the former was divided into *Professional indictors of TSRs* and *Interpersonal indicators of TSRs*. The analysis resulted in a category system with 22 main groupings: *Professional indicators of TSRs* contained thirteen categories (and seven subcategories) and *Interpersonal indicators of TSRs* contained nine categories. The category *Antecedents of TSRs* contained ten categories (with sixteen subcategories). Double or multi-coding of passages was allowed.

The coding scheme was developed through a dialogic process between two researchers. Additionally, discussions about the coding scheme with another independent researcher, who also coded some interviews, took place, which resulted in a refinement of parts of the coding scheme. After consensus was reached, one author coded the full material. Finally, in order to test the robustness of the coding process, the intercoder reliability was calculated by another researcher based on four interviews (O’Connor & Joffe, 2020,). It turned out to be satisfactory (corrected Cohen’s kappa = 0.63; Brennan & Prediger, 1981). The full coding scheme can be obtained from the authors.

## Results

### Relational dimensions that serve as indicator of the professional and interpersonal TSR (RQ 1)

#### Indicators of a professional TSR

A TSR is situated within an educational setting that requires professional behaviour from the teacher based on professional norms. Therefore, prevalent indicators of a TSR from the teachers’ perspectives belong mostly to the professional relationship sphere.

The HE teachers referred to a plethora of professional TSR characteristics. One core characteristic that was frequently mentioned by the teachers is *mutual engagement* in the TSR, reflecting its reciprocal nature:

It has to be two-way. It has to be reciprocal. So, I mean, I can give them all the time that I can give them. I can give them all the advice and help. If they don’t respond, that makes it difficult. […]. I’d like to see something in return, you know. […] So that’s probably the main thing. […] That’s part of what I call a professional relationship. [It] needs to be ... it’s a two-way thing. (Interview 9, Pos. 116-122)

On the teachers’ side, engagement and effort comprise being well organised, knowledgeable, enthusiastic about teaching, and having good classroom management skills. Relatedly, on the students’ side, the HE teachers expected them to be engaged in class, show determined effort, and achieve learning outcomes. Thereby, students and teachers can develop excellent rapport, as one teacher described:

You know, you can have a particular year group that you think, “Wow”. You know, we had a wonderful year group one year where the students were creative and dynamic and keen and professional and interested ... It was a significant enough group, it made a difference. It lifted everybody, because if they did a presentation, you know, when they were involved in a presentation, it was really high quality. It was really good stuff! (Interview 10, Pos. 207)

Conversely, the teachers listed students’ behaviours that interfere with the maintenance of a positive TSR: being late/missing class, not paying attention, being disruptive or distracted by mobile phones, or disregarding responsibilities or assignment deadlines.

Furthermore, the teachers argued that a high-quality professional relationship includes teachers’ *academic support*, *care*, and *approachability*. Helping students learn is an inherent role of the teacher. Most teachers helped students to achieve their learning goals and described themselves as facilitators. Still, the teachers emphasised that students’ interest in the offered support is equally important for TSRs; for example, if teachers noticed that their efforts in supporting students’ independence remained unacknowledged, they reduced their level of support.

*Caring behaviour*, when students are struggling, is another important feature of TSRs. From the teachers’ perspective, caring entails being willing to listen to students’ academic problems and fears and being encouraging. According to teachers, caring involves showing concern (e.g. contacting absent students). Still, the teachers frequently highlighted that their care within a TSR has its limits:

No, as far as I am concerned, I’ve done enough to try and help her. Two emails for the first assignment not submitted. Two emails for the second assignment not submitted. […] And, and, you know, really, there has to be a line, where I have to stop. (laughs) (2_Interview 8, Pos. 41)

The HE teachers also emphasised *approachability* as an inherent feature of TSRs. From their viewpoint, approachability can be achieved, for example, by having an open-door policy and being available (during/outside of office hours, before/after class, in person/online). Although the teachers considered promptly answering calls and emails important for TSRs, they also cautioned students not to be over-reliant and expect answers at any time. In addition, the teachers reflected that students should feel comfortable to meet and ask questions:

So, I think, the approachability is a practical thing, if you have time, but also a psychological one... This isn’t a person I am scared of. It’s a person who is working with me to help me to achieve these goals. (Interview 6, Pos. 89)

To achieve a positive relationship, *fairness* and *consistency/flexibility with classroom rules* (e.g. assessment and grading) also play a role. The teachers mentioned trying to treat students in a fair manner. For example, most teachers considered it unfair when they extend deadlines for individuals. The teachers reported that having clear requirements also helps to maintain good TSRs.

Naturally, a TSR is inherently unbalanced regarding *power distribution*, especially concerning assessment. The teachers suggested that this power imbalance, which can negatively affect TSRs, can be flattened through informal communication patterns and student-centeredness. Different understandings of preferred *(in)formality* in TSRs by teachers and students, which can often be traced back to their (different) cultural backgrounds, can lead to tensions in a TSR; however, the right balance can foster a relationship in which both actors feel comfortable. The teachers explained that authenticity and empathy were also advantageous in cultivating a positive TSR.

Overall, TSRs are characterised by mutuality, as in the form of reciprocal *friendliness*, *trust and honesty*, *respect and tolerance*, and *openness*. To establish positive TSRs, the teachers claimed they are friendly and polite when interrupting or correcting students, and they expect students to be friendly as well. The teachers also mentioned being honest about students’ academic behaviour and learning success. Conversely, within a TSR, the teachers also wanted the students to be honest in terms of academic integrity. By establishing possibilities for honest communication and feedback, teachers felt that they can create a trusting environment:

I think that’s how relationships are built. I think they fall down if ... if students feel that you can’t be trusted. So, if you say, you are going to do something and then don’t do it. Or if you say, they are working well, and then they fail an assignment. I think it is absolute crucial to be honest. So, if somebody … isn’t doing well, you need to actually talk to them and express your concerns. (Interview 4, Pos. 89)

According to the teachers, trust in professional TSRs can also be fostered by being reliable when making decisions (teachers) or by meeting academic responsibilities (students). To build and maintain a good TSR, both parties should also demonstrate respectful and tolerant behaviour. According to the teachers’ accounts, teachers can show respect by valuing students’ opinions and by professionally reprimanding or correcting students. Students can express respect by adhering to teachers’ rules and boundaries and by paying attention in class. When teachers embrace students’ questions and feedback, and when students are open-minded about the teaching content and methods, they can together create a tolerant, open classroom climate and a high-quality professional relationship.

#### Indicators of interpersonal TSRs

When describing TSRs in HE, most teachers referred to indicators of a professional TSR, i.e. how teachers and students shape the working relationship. However, relationships are also formed interpersonally. This is reflected, for example, in getting to know each other personally.

*Closeness* was found to be a highly salient interpersonal feature for HE teachers. While some teachers explained that they see TSRs solely in professional terms, others felt that establishing close(r) relationships is important. The teachers mentioned different ideas about how to establish close(r) bonds with students, e.g. by disclosing personal information, telling personal anecdotes, or asking about and remembering personal details of the students:

I should’ve said that before, remembering people’s names is number 1 priority and remembering things about them. So, you know, when you see them the following week. You say, “Oh, how...” ... you know, .“How did you ... how was the film?” or whatever. Or someone has had a birthday party, you know: “Did you have a good time?” ... that kind of thing. (Interview 4, Pos. 73)

The teachers had different understandings of the appropriate level of closeness, as the following examples show:

I tell my students, I give them my mobile telephone number. I trust them. … and I tell them that I wouldn’t appreciate … a call in the middle of the night. But I think that when students’ lives are very busy and that they have ... and sometimes they just want to be able to speak to somebody, I am happy to do that. […] And I feel that they respect the trust that you have in them by giving the number. (Interview 5, Pos. 53)

Relatedly, according to the teachers, close relationships require interpersonal *trust and honesty*. However, some teachers emphasised a boundary for closeness, sometimes referred to as “professional distance”:

I never give students my mobile phone number. […] I try and keep what I think of a professional distance if you like. (Interview 10, Pos. 115)

According to many HE teachers, too much closeness can become uncomfortable, and it was deemed improper or even dangerous (e.g. becoming a student’s buddy). Some teachers reflected that too much self-disclosure on either side can be awkward; therefore, according to them, teachers clearly have to set boundaries (e.g. deciding whether to become connected on social media) to carefully establish an “appropriate” interpersonal TSR.

Except for closeness, all other relational indicators were found within both dimensions of TSRs. Altogether, some teachers explained that it is natural to feel more affinity with particular students, with whom it is also easier to establish close relationships. However, the teachers considered it unfair to let their personal preferences, based on affinities, prevail. They, therefore, argued trying to hide their personal favourites (teachers’ pets) by being equally approachable and supportive for all students to maintain a *fair*, *interpersonal *TSR.

From the HE teachers’ viewpoint, interpersonal *support*, *approachability*, and *care* can also be part of a TSR. Teachers claimed that academic support is a vital facet of TSRs, and indeed, interpersonal support, i.e. helping students regarding personal matters, occurred less frequently than academic support in the accounts. Some teachers explained that students should feel comfortable to meet with them about not only academic matters but also personal ones. From their perspective, when students share their personal problems and have a “comfort chat”, this can foster an interpersonal TSR. In that regard, the teachers described interpersonal care as showing concern for students’ private issues:

And probably on a couple of occasions I’ve given the student a hug. But that’s because the tears have been ... it won’t have been about the work. The real problem would have been something going on in their lives. And sometimes the talk about an assignment has actually got nothing to do with the assignment. The talk is much more about, something terrible is happening, you know, their husband’s left them, and they just need to find a way to have somebody to talk about it. (2_Interview 3, Pos. 170)

Lastly, the HE teachers viewed mutual *respect and tolerance* and *openness* as core elements of the interpersonal TSR. They claimed that both actors should show respectful, tolerant, and open behaviour towards everyone in class, regardless of their individual background (e.g. origin or religious beliefs).

### Antecedents of TSR (RQ 2)

TSRs can develop quite differently since several factors foster or impede the building and maintenance of high-quality TSRs. Overall, two main influencing categories were determined: *contextual* and *personal factors*.

The teachers highlighted that contextual factors significantly influence TSRs. Context includes *course and university conditions*, *assignment and achievement situations*, and *cultural setting*. Regarding course conditions, the teachers mentioned several influencing sub-facets. In their opinion, it is easier to foster (closer) relationships with higher levels of approachability and support in groups with smaller *numbers of students*. Moreover, according to the teachers’ experiences, the *day of the week* or *time of the day* of the course can facilitate or hinder the establishment of positive relationships; the teachers stressed that morning (especially Monday) or late afternoon (especially Friday) classes can be challenging since students can seem less motivated.

Teachers of maths, science, and literacy, especially, saw the *subject area* as a relevant antecedent, reporting anxious students with negative preconceptions about the subject and teaching pedagogy. Students’ adverse attitudes can block their willingness to form positive TSRs:

There has been the usual difficulties. The students not liking and then displaying their dislike ... they bring their dislike with them. I thought that’s … hatred of mathematics. […] If it gets into the way of relating and if they start to think that it’s someone else’s fault and, you know, that’s where, I think, some problem might begin. (2_Interview 1, Pos. 167-169)

Apart from course conditions and factors resulting from the subject area taught, *university conditions* can also affect TSRs. The teachers mentioned that students’ experience of an *established university community on campus* can positively affect TSRs. *Pressure on university staff* (e.g. publishing pressure, teaching pressure), however, can influence the relationship negatively, as lecturers have limited time/flexibility to devote to students.

Within the HE sphere, teachers must *measure students’ performance*. The teachers reported that students sometimes dislike them or complain because of grading criteria or rules. The relationship can suffer when students receive poor grades and blame the teacher. In the worst cases, students might terminate the TSR by dropping out.

The teaching and learning context is always embedded within a multi-layered *cultural setting* in regards to regional, institutional and organisational factors. As such, the teachers highlighted that the respective culture must not be overlooked since it shapes teachers’ and students’ perceptions of “appropriate” behaviours within a TSR.

Besides those contextual, respective environmental factors, the HE teachers also claimed that *personal factors* can make a difference in TSRs.

The teachers explained different needs within the relationship through *teachers’ and students’ backgrounds*. Often, they stressed the *cultural background* of both actors; in their opinion, students from Europe or Asia prefer more formal relationships, whereas Australian students appear to be more informal and open. Teachers reported misunderstandings and clashes in the relationship when either party originates from a different cultural background and has difficulties adapting to the cultural understanding of a TSR. Moreover, *teachers’ and students’ gender*, *teachers’ type of contract*, *students’ age and perceived maturity*, *their socioeconomic status*, *health conditions*, and *status as first-generation university students* or *local or external student* can make a difference for TSRs. Regarding gender, most teachers mentioned that their own and the students’ gender makes no difference in TSRs. However, male students were sometimes described as less talkative and lacking in motivation, although some teachers reported that they can relate to them more easily because they seemed more easy-going. Age and perceived maturity can have different effects on the establishment of a TSR. The teachers often described younger students as more immature, peer-conscious, unprofessional, and challenging to engage compared to older/more mature students, who were perceived as working harder and being more independent. Although teachers claimed that students’ different socioeconomic status, their health conditions (mental or physical), and their status as first-generation university students make no fundamental difference on TSRs, they tried to provide more support within a TSR to those students who are underserved.

The teachers also mentioned a difference in TSR between *external and campus students:* building relationships with externals sometimes requires more effort. *Teachers’ position at university* can also affect the relationship. For example, sessional lecturers, i.e. academic staff that usually has short-term contracts and is solely responsible for teaching, claimed that they cannot support students as much because they are not on campus as often as regular academic staff.

The realisation of good TSRs also depends on *teachers’ and students’ personalities*. The HE teachers reported difficulties in forming relationships with overly shy, quiet students, as they are harder to approach. On the other hand, the teachers mentioned that students who exhibited too high a level of extraversion (e.g. too much dominance) or who showed too much self-disclosure could be annoying, which also hindered the establishment of a high-quality TSR. However, the teachers were also mindful that not all students in class may appreciate the teacher’s personality:

So, some people will think that you’re corny, or you are dour, or you’re boring. And you can’t possibly please those people. Some people will be annoyed with you.... You know, the way that you get distracted, and you try and put time into making them feel socially warm and accepted in an environment. […] Other people will be appreciative, and they’ll say, “Oh, he makes me feel comfortable. I feel really, you know, secure and ... and I am not stressed. […] So, it depends on our brain and our personalities. (Interview 12, Pos. 117)

Due to teachers’ and students’ different personalities and their interplay in class, certain *classroom dynamics* can arise, which foster or hinder the establishment of positive TSRs. Some of the teachers described that different groups of students trigger different classroom dynamics in the form of an energetic or challenging classroom atmosphere. For example, they reported that the absence or presence of single students in class can make a significant difference in shaping the TSR with a group. Maintaining an open and honest *communication* when interacting in the classroom also contributes to the formation of a positive TSR. As such, some of the teachers mentioned that the underlying tone and meaning that is conveyed through students’ utterances also influences TSR.

Lastly, similarity plays a major facilitating role in building high-quality TSRs. Similar backgrounds (e.g. teacher and students sharing same/similar age, cultural background, and family situation), personality traits, or hobbies/common interests support the establishment of (close) relationships, as a teacher explained:

You have a closeness with people because you have got something in your personality that’s the same. You might share the same humour or, you know, have the same sort of philosophy background. So, you do develop ... some sort of closeness, but still, there is a line. (Interview 11, Pos. 71)

**Fig. 1:**
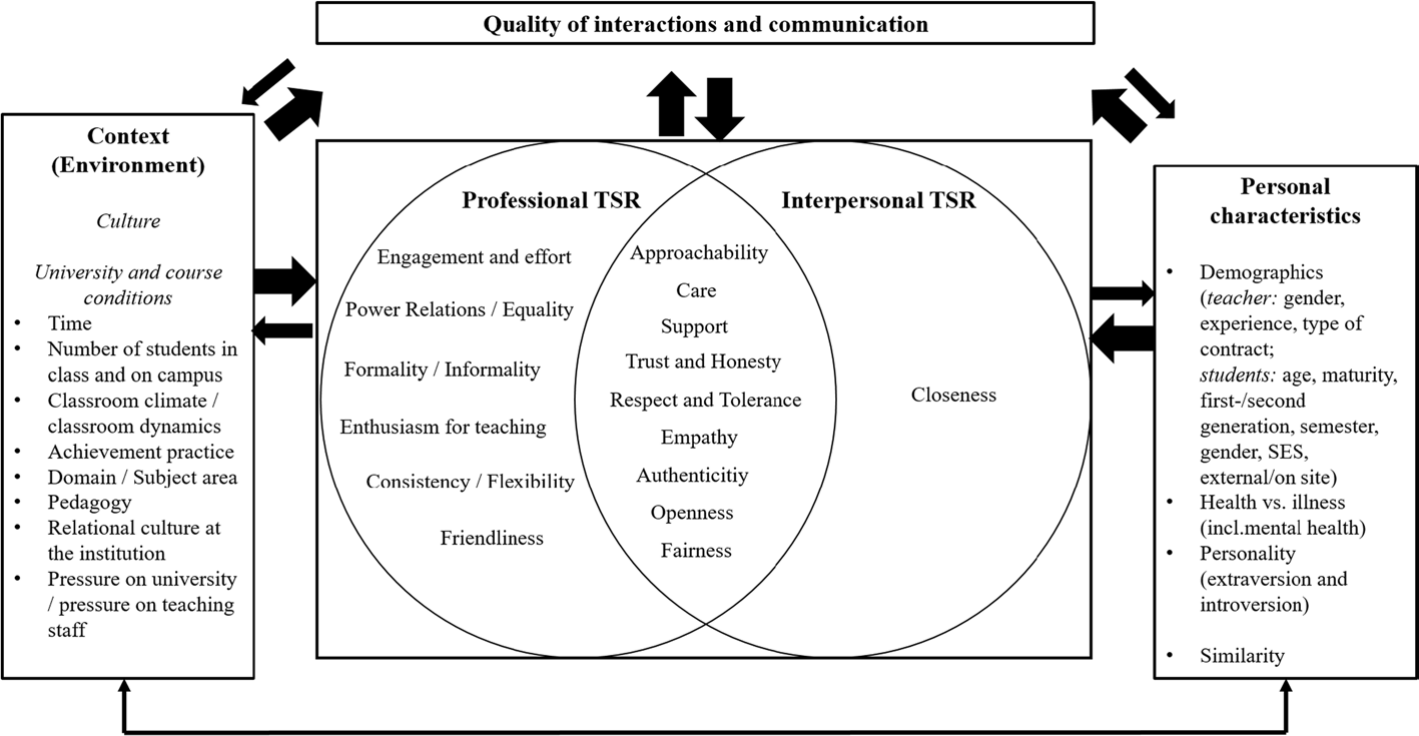
Conceptual model on TSRs in HE — an overview

As a synthesis of the relevant relational dimensions and their influences resulting from the analysis of the interviews, we propose the following first tentative conceptual model for TSRs in HE (see Fig. 1). To summarise, the model illustrates the multidimensional nature of TSRs, with several relational facets comprised within each dimension. In addition, it takes into account that several environmental and personal characteristics, as well as the quality of the concrete interactions, contribute to the development of a TSR. Finally, if we conceptualise TSRs as dynamic and processual, the constitutive elements and causes of TSRs are strongly intertwined.

## Discussion

This study aimed to investigate the perspectives of HE teachers on the relational indicators and influencing factors of a positive relationship with (first-year) students in initial teacher education.

Although not explicitly addressed in the interview questions, the teachers’ statements confirmed the multidimensional nature of the TSR, distinguishing between the professional and the interpersonal facets (Hagenauer & Volet, 2014). These two dimensions have unique features, but they also show a high overlap in the core characteristics. For example, support can be provided on the academic/professional level as well as on the interpersonal/private level, paralleling the findings on schoolteachers’ social support, which distinguish between the academic (e.g. informational) support and the emotionally related support of teachers (Lei et al., 2018). Nonetheless, the teachers in this study emphasised the professional dimension of the TSR more strongly than the interpersonal component, a finding that was also reported by Eloff et al. (2021), who showed that HE students addressed academic-related issues more frequently with the HE teachers’ role than personal ones. However, this finding should not lead to the conclusion that the professional aspects of the relationship may be more important for having a successful college experience. For example, Snijders et al. (2020) have shown that affective commitment, as an indicator of the interpersonal dimension, had more powerful effects on students’ engagement than stronger professionally related components of a TSR. Thus, the higher prevalence of issues related to the professional side of TSRs in the present study may be explained by the fact that, overall, the professional role dominates in the interactions between teachers and students.

Another explanation would be that it is easier to talk openly about aspects related to the professional role as a teacher compared to interpersonal and more private issues. It remains to be seen how the strengths of the effects of the professional and the interpersonal dimensions unfold in direct comparison. Future research that applies a quantitative methodology may have to overcome challenges when testing comparative effects statistically as we have detected a high interdependency in the relational indicators of TSRs, suggesting a significant amount of shared variance in statistical terms.

Pertaining to the professional dimension of TSR, it has to be mentioned that the teachers in this study have all taught in the field of teacher education, which is a specific context in HE that may also affect the perception of teacher professionalism. It needs to be further explored if similar responses regarding professionalism in the TSR are given in other disciplines in HE (see for example Lindblom-Ylänne et al., 2006).

Furthermore, mutuality was mentioned very frequently in respect to different relational indicators by the HE teachers, emphasising reciprocity as a core dimension of TSRs. As a result, there is a significant overlap of indicators of high-quality relationships between the accounts of the students, as found in previous studies (e.g. Anderson et al., 2020; Denzine & Pulos, 2000; Dicker et al., 2019; Dobransky & Frymier, 2004; Snijeders et al., 2021), and the findings of the present study from the teachers’ perspective.

In addition, the results unveiled that the TSR is characterised by multiple boundaries that need to be maintained. For example, teachers need to provide academic support to their students, but simultaneously, this support needs to be at a level to fruitfully foster students’ self-regulated learning. The appropriateness will vary, depending on whether the students are in their first year or already advanced in their studies, suggesting that some student populations may need more support (guidance) than others. This assumption aligns well with findings of Hassel and Ridout (2018), who showed that teachers adapt their teaching to a more transmission-oriented style when teaching first-year students, while they teach more student-centred with more advanced students. Relatedly, self-disclosure as a facet of the interpersonal dimension is important to stimulate closeness between students and teachers, but self-disclosure needs to stay within professional boundaries (Holmes et al., 1999) .

Finally, the results showed that the quality of TSRs strongly depends on various personal and contextual factors. Culture as an influencing factor was very salient in the HE teachers’ accounts, suggesting that the perceived appropriateness of teacher-student interactions depends greatly on cultural-educational background (Volet, 2001).

### Study limitations and future research

This study brings to light teachers’ reflections on the quality and antecedents of TSRs in HE. As with every study, this one comes with certain limitations. First, the generalizability of the findings is limited as the data were from HE teachers in one country, all in the field of teacher education, and with first-year students only. Second, as already addressed, interpersonal issues in the TSRs may have been underestimated compared to the professional aspects. Third, although we have tried to establish an atmosphere of collegiality, socially desirable responding may have not been fully ruled out. Finally, the data was collected in 2012. Aspects on how TSRs may be established in digitalized learning environments or how digitalization affects TSRs was not a core focus of the study. This area would most likely be addressed much more if the study had been conducted in the last few years.

Considering these limitations, future studies need to include other contexts in order to explore the similarities but also the differences with regard to TSRs from disparate circumstances. In addition, digitalized learning environments need to be systematically compared to experiences in face-to-face learning environments with regard to TSRs. Furthermore, besides measures on the subjective view of TSRs, bringing in the perspectives of independent observers in assessing the quality of teacher-student interactions would be highly interesting as well. Having said that, as the TSR is characterised by mutuality, the students’ perspective would add another important insight for the description of the relationship. Finally, future studies should test how aspects of the interpersonal relationship and aspects of the professional relationship influence various students’ outcomes; and again, it seems important that more attention is also paid to the relation between a TSR and teachers’ positive experiences, such as their occupational well-being or their engagement in teaching.

### Practical implications

We have shown that the establishment of a high-quality TSR in HE is very complex due to its multidimensional and dynamic/reciprocal nature, one influenced by and influencing various contextual and personal factors as well as concrete student–teacher interactions. Regarding the multidimensionality of TSRs, it seems important that teachers increase the clarity of their role as regards the professional and the interpersonal dimensions a HE teacher wants to bring into this relationship. If role clarity can be achieved or at least enhanced, as well as clarity regarding the expectations that are bound to these roles, a deliberate establishment of high-quality relationships may be fostered. As a TSR is reciprocal and responsive, role clarity does not only need to be established on the teachers’ side but also on the students’ (see also Naylor et al., 2021). This is especially true for first-year students, who have to adapt to a new learning-teaching environment, which typically expects more independent and self-regulated learning from them compared to the school context (Hassel & Ridout, 2018). Furthermore, as the development of a TSR is a highly individualised process, relying on concrete interactions with students and groups of students bringing in different personalities and backgrounds, HE teachers need to possess strong interpersonal skills in order to build and maintain positive relationships in an adaptive manner. Continuous professional development addressing the interpersonal skills of HE teachers may contribute to the establishment of high-quality TSRs.

Simultaneously, contextual conditions need to be provided that promote high-quality TSRs, for example, small-group teaching, spaces where HE teachers and students can meet and, last but not least, a reasonable teaching load that allows interacting with students closely. As indicators, such as approachability, support and care are at the core of a positive TSR, providing a HE environment that allows students and teachers to interact with each other regularly and sufficiently seems to be of utmost importance. It is open to debate how, in times of the massification of HE, with an increased focus on research output, the promotion of quality TSRs can be sufficiently fostered, so that these relationships can trigger not only rich teaching and learning experiences, but also the expected positive effects on teachers’ and students’ well-being. Finally, digitalization in HE teaching and learning has received increased importance (e.g. Sharma et al., 2022). Consequently, concepts and strategies have to be developed for establishing and supporting positive TSRs in digitalized learning environments.
